# (*E*)-*N*′-(2-Hy­droxy-3,5-diiodo­benzyl­idene)-3-methyl­benzohydrazide

**DOI:** 10.1107/S160053681200387X

**Published:** 2012-02-04

**Authors:** Chun-Bao Tang

**Affiliations:** a Department of Chemistry, Jiaying University, Meizhou 514015, People’s Republic of China

## Abstract

In the title compound, C_15_H_12_I_2_N_2_O_2_, the dihedral angle between the benzene rings is 26.5 (3)° and the mol­ecule has an *E* configuration about the C=N bond. An intra­molecular O—H⋯N hydrogen bond is observed in the mol­ecule. In the crystal, mol­ecules are linked by N—H⋯O hydrogen bonds, forming chains along the *c* axis.

## Related literature

For general background to hydrazones, see: Rasras *et al.* (2010 ▶); Pyta *et al.* (2010 ▶); Angelusiu *et al.* (2010 ▶). For related structures, see: Fun *et al.* (2008 ▶); Singh & Singh (2010 ▶); Ahmad *et al.* (2010 ▶); Tang (2010 ▶, 2011 ▶). For reference bond-length data, see: Allen *et al.* (1987 ▶).
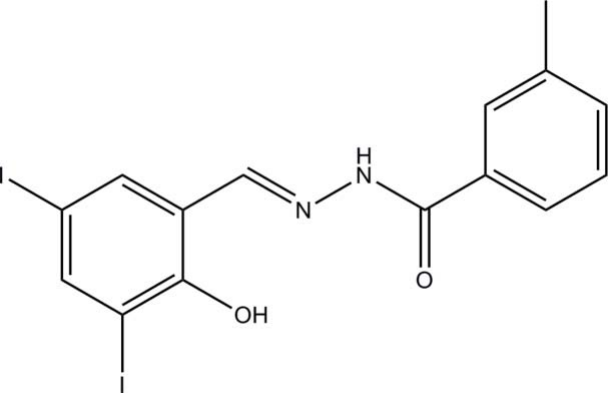


## Experimental

### 

#### Crystal data

C_15_H_12_I_2_N_2_O_2_
*M* *_r_* = 506.07
Monoclinic, 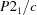

*a* = 14.778 (3) Å
*b* = 11.764 (3) Å
*c* = 9.8480 (19) Å
β = 102.191 (2)°
*V* = 1673.4 (6) Å^3^
*Z* = 4
Mo *K*α radiation
μ = 3.76 mm^−1^
*T* = 298 K
0.17 × 0.15 × 0.15 mm

#### Data collection

Bruker SMART CCD area-detector diffractometer
Absorption correction: multi-scan (*SADABS*; Sheldrick, 1996 ▶) *T*_min_ = 0.567, *T*_max_ = 0.602
11923 measured reflections
3430 independent reflections
2072 reflections with *I* > 2σ(*I*)
*R*_int_ = 0.045

#### Refinement

*R*[*F* ^2^ > 2σ(*F* ^2^)] = 0.069
*wR*(*F* ^2^) = 0.175
*S* = 1.02
3430 reflections
196 parameters
1 restraint
H atoms treated by a mixture of independent and constrained refinement
Δρ_max_ = 2.01 e Å^−3^
Δρ_min_ = −1.21 e Å^−3^

### 

Data collection: *SMART* (Bruker, 2002 ▶); cell refinement: *SAINT* (Bruker, 2002 ▶); data reduction: *SAINT*; program(s) used to solve structure: *SHELXS97* (Sheldrick, 2008 ▶); program(s) used to refine structure: *SHELXL97* (Sheldrick, 2008 ▶); molecular graphics: *SHELXTL* (Sheldrick, 2008 ▶); software used to prepare material for publication: *SHELXL97*.

## Supplementary Material

Crystal structure: contains datablock(s) global, I. DOI: 10.1107/S160053681200387X/su2373sup1.cif

Structure factors: contains datablock(s) I. DOI: 10.1107/S160053681200387X/su2373Isup2.hkl

Supplementary material file. DOI: 10.1107/S160053681200387X/su2373Isup3.cml

Additional supplementary materials:  crystallographic information; 3D view; checkCIF report

## Figures and Tables

**Table 1:** Hydrogen-bond geometry (Å, °)

*D*—H⋯*A*	*D*—H	H⋯*A*	*D*⋯*A*	*D*—H⋯*A*
O1—H1⋯N1	0.82	1.85	2.572 (8)	146
N2—H2⋯O2^i^	0.90 (1)	1.93 (2)	2.800 (9)	162 (6)

Symmetry code: (i) 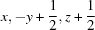.

**Fig. 1.**
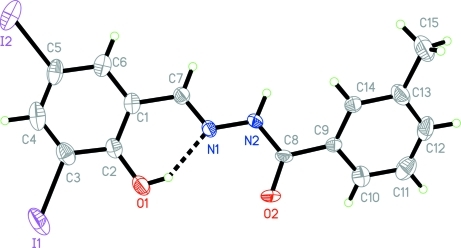
The molecular structure of the title compound, showing the atom-numbering and displacement ellipsoids drawn at the 30% probability level. The intramolecular O—H···N hydrogen bond is shown as a dashed line (see Table 1 for details).

**Fig. 2.**
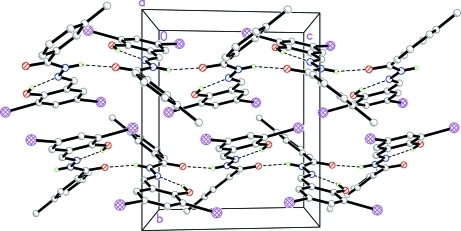
Crystal packing of the title compound, viewed along the a axis. Hydrogen bonds are shown as dashed lines (see Table 1 for details).

## supplementary crystallographic information

### Comment

Hydrazone compounds have received much attention in biological and structural chemistry in the last few years (Rasras *et al.*, 2010; Pyta *et al.*, 2010; Angelusiu *et al.*, 2010; Fun *et al.*, 2008; Singh & Singh, 2010; Ahmad *et al.*, 2010). As a continuation of our work on the structural study on such compounds (Tang, 2010, 2011), the author reports herein the crystal structure of the new title hydrazone compound.

In the title compound (Fig. 1), the dihedral angle between the two benzene rings is 26.5 (3)°. An intramolecular O1—H1···N1 hydrogen bond (Table 1) is observed in the molecule, which has an E configuration about the N1═C7 bond. Bond lengths in the compound are normal (Allen *et al.*, 1987) and comparable to those in the similar compounds mentioned above. In the crystal, molecules are linked through intermolecular N—H···O hydrogen bonds, forming chains along the *c* axis (Fig. 2 and Table 1).

### Experimental

2-Hydroxy-3,5-diiodobenzaldehyde (0.1 mmol, 37.5 mg) and 3-methylbenzohydrazide (0.1 mmol, 15.0 mg) were dissolved in methanol (20 ml). The mixture was stirred at reflux for 10 min to give a clear colourless solution. Colourless needle-shaped crystals of the compound were formed by slow evaporation of the solvent over several days.

### Refinement

The amino H atom was located in a difference Fourier map and refined isotropically, with the N—H distance restrained to 0.90 (1) Å. The other H atoms were included in calculated positions and refined as riding atoms: O—H = 0.82 Å, Csp^2^—H = 0.93 Å, and C(methyl)—H = 0.96 Å, with U_iso_(H) = k × U_eq_(O,C), where k = 1.5 for OH and CH_3_ H-atoms, and k = 1.2 for all other H-atoms.

### Crystal data

**Table:**

C_15_H_12_I_2_N_2_O_2_	*F*(000) = 952
*M**_r_* = 506.07	*D*_x_ = 2.009 Mg m^−^^3^
Monoclinic, *P*2_1_/*c*	Mo *K*α radiation, λ = 0.71073 Å
Hall symbol: -P 2ybc	Cell parameters from 2661 reflections
*a* = 14.778 (3) Å	θ = 2.2–24.4°
*b* = 11.764 (3) Å	µ = 3.76 mm^−^^1^
*c* = 9.8480 (19) Å	*T* = 298 K
β = 102.191 (2)°	Cut from needle, colourless
*V* = 1673.4 (6) Å^3^	0.17 × 0.15 × 0.15 mm
*Z* = 4	

### Data collection

**Table:**

Bruker SMART CCD area-detector diffractometer	3430 independent reflections
Radiation source: fine-focus sealed tube	2072 reflections with *I* > 2σ(*I*)
Graphite monochromator	*R*_int_ = 0.045
ω scans	θ_max_ = 26.5°, θ_min_ = 2.2°
Absorption correction: multi-scan (*SADABS*; Sheldrick, 1996)	*h* = −16→18
*T*_min_ = 0.567, *T*_max_ = 0.602	*k* = −14→14
11923 measured reflections	*l* = −12→12

### Refinement

**Table:**

Refinement on *F*^2^	Primary atom site location: structure-invariant direct methods
Least-squares matrix: full	Secondary atom site location: difference Fourier map
*R*[*F*^2^ > 2σ(*F*^2^)] = 0.069	Hydrogen site location: inferred from neighbouring sites
*wR*(*F*^2^) = 0.175	H atoms treated by a mixture of independent and constrained refinement
*S* = 1.02	*w* = 1/[σ^2^(*F*_o_^2^) + (0.058*P*)^2^ + 13.9544*P*] where *P* = (*F*_o_^2^ + 2*F*_c_^2^)/3
3430 reflections	(Δ/σ)_max_ < 0.001
196 parameters	Δρ_max_ = 2.01 e Å^−^^3^
1 restraint	Δρ_min_ = −1.21 e Å^−^^3^

### Special details

**Table:**

Geometry. All esds (except the esd in the dihedral angle between two l.s. planes) are estimated using the full covariance matrix. The cell esds are taken into account individually in the estimation of esds in distances, angles and torsion angles; correlations between esds in cell parameters are only used when they are defined by crystal symmetry. An approximate (isotropic) treatment of cell esds is used for estimating esds involving l.s. planes.
Refinement. Refinement of F^2^ against ALL reflections. The weighted R-factor wR and goodness of fit S are based on F^2^, conventional R-factors R are based on F, with F set to zero for negative F^2^. The threshold expression of F^2^ > 2sigma(F^2^) is used only for calculating R-factors(gt) etc. and is not relevant to the choice of reflections for refinement. R-factors based on F^2^ are statistically about twice as large as those based on F, and R- factors based on ALL data will be even larger.

### Fractional atomic coordinates and isotropic or equivalent isotropic displacement parameters (Å^2^)

**Table:**

	*x*	*y*	*z*	*U*_iso_*/*U*_eq_	
I1	0.35741 (6)	0.46000 (8)	0.09441 (9)	0.0923 (4)	
I2	0.26264 (6)	0.40585 (9)	0.65569 (12)	0.0979 (4)	
N1	0.6613 (5)	0.2920 (6)	0.4785 (7)	0.0399 (17)	
N2	0.7486 (5)	0.2539 (6)	0.5352 (7)	0.0410 (17)	
O1	0.5439 (5)	0.3715 (6)	0.2693 (6)	0.0589 (18)	
H1	0.5930	0.3439	0.3106	0.088*	
O2	0.7851 (4)	0.2492 (7)	0.3260 (6)	0.0599 (19)	
C1	0.5105 (6)	0.3476 (7)	0.4956 (10)	0.044 (2)	
C2	0.4846 (6)	0.3768 (7)	0.3551 (9)	0.045 (2)	
C3	0.3942 (7)	0.4123 (9)	0.3033 (11)	0.059 (3)	
C4	0.3331 (7)	0.4219 (8)	0.3891 (13)	0.066 (3)	
H4	0.2733	0.4478	0.3532	0.080*	
C5	0.3581 (6)	0.3939 (9)	0.5277 (13)	0.061 (3)	
C6	0.4465 (6)	0.3558 (8)	0.5815 (11)	0.055 (2)	
H6	0.4636	0.3355	0.6747	0.066*	
C7	0.6045 (6)	0.3093 (7)	0.5559 (9)	0.043 (2)	
H7	0.6225	0.2976	0.6512	0.052*	
C8	0.8065 (6)	0.2322 (7)	0.4513 (8)	0.039 (2)	
C9	0.8987 (6)	0.1848 (7)	0.5190 (8)	0.0377 (19)	
C10	0.9737 (7)	0.2119 (9)	0.4621 (11)	0.059 (3)	
H10	0.9661	0.2599	0.3855	0.070*	
C11	1.0589 (8)	0.1685 (11)	0.5179 (14)	0.075 (3)	
H11	1.1091	0.1863	0.4787	0.090*	
C12	1.0708 (7)	0.0988 (11)	0.6311 (12)	0.072 (3)	
H12	1.1297	0.0711	0.6691	0.086*	
C13	0.9977 (8)	0.0684 (10)	0.6908 (10)	0.067 (3)	
C14	0.9116 (7)	0.1147 (8)	0.6307 (9)	0.049 (2)	
H14	0.8610	0.0968	0.6689	0.059*	
C15	1.0119 (11)	−0.0117 (13)	0.8132 (13)	0.108 (5)	
H15A	1.0718	0.0011	0.8714	0.162*	
H15B	1.0078	−0.0888	0.7805	0.162*	
H15C	0.9650	0.0015	0.8656	0.162*	
H2	0.767 (4)	0.239 (6)	0.626 (2)	0.018 (17)*	

### Atomic displacement parameters (Å^2^)

**Table:**

	*U*^11^	*U*^22^	*U*^33^	*U*^12^	*U*^13^	*U*^23^
I1	0.0839 (6)	0.0968 (7)	0.0776 (6)	0.0291 (5)	−0.0248 (4)	0.0073 (5)
I2	0.0542 (5)	0.1061 (8)	0.1439 (9)	−0.0029 (5)	0.0449 (5)	−0.0132 (6)
N1	0.037 (4)	0.044 (4)	0.036 (4)	0.009 (3)	0.001 (3)	0.002 (3)
N2	0.041 (4)	0.056 (5)	0.024 (3)	0.010 (3)	0.002 (3)	0.001 (3)
O1	0.054 (4)	0.072 (5)	0.044 (4)	0.016 (4)	−0.005 (3)	0.002 (3)
O2	0.058 (4)	0.101 (5)	0.023 (3)	0.011 (4)	0.014 (3)	0.005 (3)
C1	0.039 (5)	0.033 (4)	0.058 (6)	−0.001 (4)	0.003 (4)	−0.008 (4)
C2	0.044 (5)	0.040 (5)	0.046 (5)	0.006 (4)	−0.002 (4)	−0.013 (4)
C3	0.061 (6)	0.053 (6)	0.056 (6)	0.008 (5)	−0.007 (5)	−0.012 (5)
C4	0.043 (6)	0.045 (6)	0.101 (9)	0.005 (5)	−0.008 (6)	−0.015 (6)
C5	0.035 (5)	0.053 (6)	0.093 (8)	−0.007 (5)	0.008 (5)	−0.013 (6)
C6	0.044 (5)	0.049 (6)	0.071 (7)	0.000 (5)	0.012 (5)	−0.007 (5)
C7	0.044 (5)	0.044 (5)	0.041 (5)	0.006 (4)	0.007 (4)	−0.004 (4)
C8	0.045 (5)	0.049 (5)	0.020 (4)	−0.001 (4)	0.006 (3)	−0.002 (3)
C9	0.040 (5)	0.045 (5)	0.027 (4)	0.003 (4)	0.006 (3)	−0.006 (4)
C10	0.052 (6)	0.058 (6)	0.064 (6)	0.002 (5)	0.007 (5)	0.000 (5)
C11	0.049 (7)	0.090 (9)	0.091 (9)	−0.008 (6)	0.028 (6)	−0.011 (7)
C12	0.039 (6)	0.101 (9)	0.070 (8)	0.017 (6)	0.000 (5)	−0.025 (7)
C13	0.074 (8)	0.078 (8)	0.042 (5)	0.027 (6)	−0.008 (5)	−0.002 (5)
C14	0.051 (5)	0.065 (6)	0.032 (5)	0.015 (5)	0.009 (4)	−0.008 (4)
C15	0.114 (11)	0.133 (13)	0.069 (8)	0.070 (10)	0.001 (8)	0.016 (8)

### Geometric parameters (Å, º)

**Table:**

I1—C3	2.090 (10)	C6—H6	0.9300
I2—C5	2.085 (11)	C7—H7	0.9300
N1—C7	1.264 (10)	C8—C9	1.493 (11)
N1—N2	1.368 (9)	C9—C14	1.356 (12)
N2—C8	1.334 (10)	C9—C10	1.381 (13)
N2—H2	0.897 (10)	C10—C11	1.362 (15)
O1—C2	1.341 (11)	C10—H10	0.9300
O1—H1	0.8200	C11—C12	1.365 (17)
O2—C8	1.224 (9)	C11—H11	0.9300
C1—C2	1.399 (12)	C12—C13	1.381 (16)
C1—C6	1.399 (13)	C12—H12	0.9300
C1—C7	1.461 (12)	C13—C14	1.395 (13)
C2—C3	1.390 (13)	C13—C15	1.510 (16)
C3—C4	1.365 (15)	C14—H14	0.9300
C4—C5	1.377 (16)	C15—H15A	0.9600
C4—H4	0.9300	C15—H15B	0.9600
C5—C6	1.377 (13)	C15—H15C	0.9600
			
C7—N1—N2	119.7 (7)	O2—C8—C9	121.9 (8)
C8—N2—N1	118.8 (6)	N2—C8—C9	116.1 (6)
C8—N2—H2	119 (4)	C14—C9—C10	118.9 (8)
N1—N2—H2	123 (4)	C14—C9—C8	123.2 (8)
C2—O1—H1	109.5	C10—C9—C8	117.9 (8)
C2—C1—C6	120.2 (8)	C11—C10—C9	120.1 (10)
C2—C1—C7	121.0 (8)	C11—C10—H10	119.9
C6—C1—C7	118.8 (8)	C9—C10—H10	119.9
O1—C2—C3	119.2 (8)	C10—C11—C12	120.3 (11)
O1—C2—C1	122.2 (8)	C10—C11—H11	119.9
C3—C2—C1	118.5 (9)	C12—C11—H11	119.9
C4—C3—C2	120.5 (10)	C11—C12—C13	121.7 (10)
C4—C3—I1	121.2 (8)	C11—C12—H12	119.2
C2—C3—I1	118.1 (8)	C13—C12—H12	119.2
C3—C4—C5	121.3 (10)	C12—C13—C14	116.4 (10)
C3—C4—H4	119.3	C12—C13—C15	120.8 (11)
C5—C4—H4	119.3	C14—C13—C15	122.8 (11)
C6—C5—C4	119.6 (10)	C9—C14—C13	122.7 (10)
C6—C5—I2	119.8 (9)	C9—C14—H14	118.6
C4—C5—I2	120.6 (8)	C13—C14—H14	118.6
C5—C6—C1	119.8 (10)	C13—C15—H15A	109.5
C5—C6—H6	120.1	C13—C15—H15B	109.5
C1—C6—H6	120.1	H15A—C15—H15B	109.5
N1—C7—C1	120.0 (8)	C13—C15—H15C	109.5
N1—C7—H7	120.0	H15A—C15—H15C	109.5
C1—C7—H7	120.0	H15B—C15—H15C	109.5
O2—C8—N2	122.1 (8)		

### Hydrogen-bond geometry (Å, º)

**Table:**

*D*—H···*A*	*D*—H	H···*A*	*D*···*A*	*D*—H···*A*
O1—H1···N1	0.82	1.85	2.572 (8)	146
N2—H2···O2^i^	0.90 (1)	1.93 (2)	2.800 (9)	162 (6)

Symmetry code: (i) *x*, −*y*+1/2, *z*+1/2.
